# Differences in Practice Patterns and Payments for Female and Male Dermatologists: A Canadian Population-Based Study Over 3 Decades

**DOI:** 10.1177/12034754221119500

**Published:** 2022-09-05

**Authors:** Jorge R. Georgakopoulos, Tina Felfeli, Mayilee Canizares, Ya-Ping Jin, Marissa Joseph, Jensen Yeung, Yvonne M. Buys

**Affiliations:** 1 7938 Division of Dermatology, Department of Medicine, University of Toronto, Ontario, Canada; 2 7938 Department of Ophthalmology and Vision Sciences, University of Toronto, Ontario, Canada; 3 7938 Dalla Lana School of Public Health, University of Toronto, Ontario, Canada; 4 7989 Schroeder Arthritis Institute, Krembil Research Institute, Toronto Western Hospital, University Health Network, Ontario, Canada; 5 7938 Women’s College Hospital, Toronto, Ontario, Canada; 6 7938 Sunnybrook Health Sciences Centre, Toronto, Ontario, Canada

**Keywords:** dermatology, population-based study, practice patterns, workload, sex, female representation

## Abstract

**Background:**

Canada’s fee-for-service physician reimbursement system, where a set rate is provided for each service, suggests that a physician sex pay gap should not exist. However, recent evidence has questioned this presumption.

**Objectives:**

To characterize trends in demographics and billing, overall and by sex, for dermatologists compared to other medical and surgical specialty groups in Ontario, Canada.

**Methods:**

Using population-based data, analysis of physician billing and clinical activity from Ontario, Canada, over 27 years (1992-2018) was performed. Multilevel regression models were used to examine unadjusted and adjusted differences in payments between females and males over time, while controlling for age, distinct patients seen, patient visits, and full-time equivalent.

**Results:**

A total of 22 389 physicians were included in the analyses, including 381 dermatologists. The proportion of female dermatologists increased from 32% in 1992 to 46% in 2018. Dermatologists’ median Ontario Health Insurance Plan (OHIP) payments were $415 340 (IQR: 285 630-566 580) in 1992 compared to $296 750 (IQR: 164 480-493 180) in 2018. Male dermatologists’ OHIP payments were 20% more than their female counterparts across the entire study period. After adjusting for practice volumes, there was no significant pay gap amongst female and male dermatologists (*P* = .42); however, the sex pay gap remained significant for the other specialty groups (*P* < .001). From 1992 to 2018, dermatologists on average saw 19% fewer distinct patients per year and 15% fewer visits per patient.

**Conclusions:**

The overall sex pay gap within medical dermatology can be attributed to differences in practice patterns, whereas the sex pay gap remained significant in the other specialty groups.

## Introduction

There has been a notable change in the demographics of physicians over the past decade with proportionate female representation increasing in medical schools, residencies, and among practicing physicians.^1-3^ Previous publications have highlighted the increasing representation of females in traditionally male-dominated specialties.^4^ However, trends in female representation within dermatology, which is often viewed as a specialty with a smaller sex imbalance, have not been well explored. These data are further limited within the Canadian literature, where changes in Canadian dermatologic demographics are restricted to short-term population-based studies.^5^ As the proportion of females entering medical school increases, addressing potential organizational inequalities may help improve the appeal of certain specialties for future generations of female physicians.^6,7^

Income is often at the forefront of sex-based analysis amongst medical specialties.^8,9^ This is because a physician sex pay gap is not expected to exist specifically in those countries where physicians’ income is based on fee-for-service—a set rate applied to each service provided regardless of age, sex, or any other unique characteristics. However, recent studies comparing gross clinical payments between female and male physicians have found a notable pay gap.^10,11^ Similar disparities in remuneration of females and males have been also noted amongst other surgical and medical specialties in several countries during the last decade.^12-18^ The pay gap is evident despite the growing feminization of the physician workforce over the past decades.^1^ It has been argued that these differences in part may be explained by specialization, work hours, and productivity,^19^ which may misleadingly provide justification for these outcomes.

The province of Ontario has the largest cohort of medical dermatologists in Canada receiving fee-for-service payment through the Ontario Health Insurance Plan (OHIP). To our knowledge, no studies to date have described the trends in numbers, demographics, and billing of practicing dermatologists. We aimed to explore the potential sex pay gap in dermatology and how a change in the demographics of dermatologists has impacted the practice patterns and billing trends within the specialty.

## Methods

### Data Source

This was a population-based study using health administrative data held at IC/ES, an independent, nonprofit research institute. All available physician demographics and billings from OHIP payments from 1992 to 2018 were included. This database includes all fee-for-service payments where physicians bill for the services they provide using predefined fee service codes.^20^ One of the major advantages of the OHIP billings database is that it includes province-wide data on all physicians in hospital and outpatient settings including their demographics, clinical activity, and breadth of practice. The ICES administrative database uses information on biologic females and males at birth, as such sex was used to present the reported findings. This project was approved by the Research Ethics Board at University Health Network, Toronto, Canada.

### Physician Specialty Groups

Physician specialty is defined within the database based on specific identification codes. In addition to dermatology, other specialties were categorized into one of three groups including medical nonprocedural, medical procedural, and surgical.^21^ Medical nonprocedural specialty group included general internal medicine, clinical immunology, endocrinology, geriatrics, hematology, medical oncology, neurology, pediatrics, physical medicine and rehabilitation, psychiatry, and rheumatology. Medical procedural specialty group included cardiology, gastroenterology, nephrology, radiation oncology, and respirology. Surgical specialty group included cardiac and thoracic surgery, general surgery including pediatric general surgery, neurosurgery, obstetrics and gynecology, ophthalmology, orthopedic surgery, otolaryngology, plastic surgery, urology, and vascular surgery.

### Outcome Measures

Reported outcomes included yearly number of dermatologists, median OHIP payments, number of distinct patients seen, number of patient visits, and dermatologist sex and age. Data were also analyzed using full-time equivalent (FTE) as a measure of workload, calculated using physician yearly payments; 1 FTE was assigned to physician payments between the 40th and 60th percentiles, <1 FTE to below the 40th percentile and >1 FTE to above the 60th percentile.^22^ The main outcome measure was yearly median OHIP billings for dermatologists over time based on sex, while using age, number of unique patients in the practice, patient visits, and visits per patient as covariates. Sex differences were compared to other medical nonprocedural, medical procedural and surgical specialty groups. For all years included in the analysis, the reported dollars were converted to 2018 Canadian dollars.

### Statistical Analysis

Data were reported descriptively for the yearly number of practicing physicians, median payments, number of unique patients, and visits per patient for male and female physicians in each of the specialty groups. For determining trends over time, percent change calculations were used. Percent differences were calculated for differences between females to males and each of the specialty groups. In addition, the proportionate distribution by sex and FTE was compared. Ratio of female to male median payments were compared. The median and percentiles of the distribution for age, payments, number of unique patients, and visits per patient were calculated overall and by FTE groups by sex and specialty group for the most recent year of data (2018). Multilevel regression models were used to examine unadjusted and adjusted differences in payments between females and males over time, while controlling for age, number of unique patients seen, and number of patient visits. Interactions between year and sex were included in the models to examine if the sex gap varied over time. Multilevel models allow accounting for autocorrelation within physicians for this type of data. SAS version 9.4 was used to perform all analyses.

## Results

**Figure 1:**
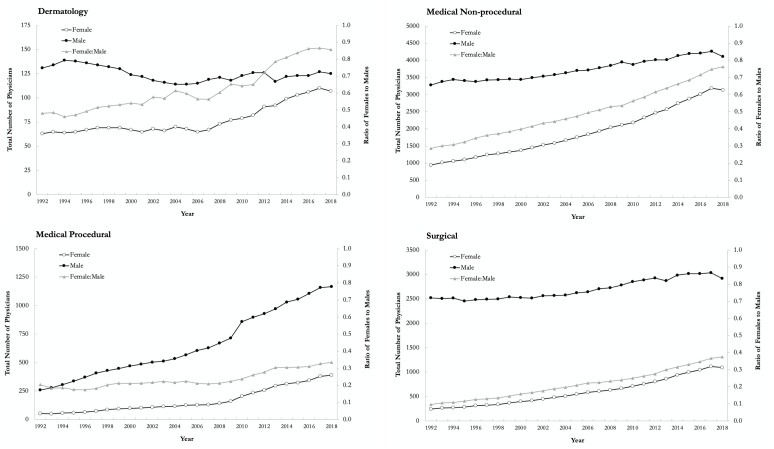
Yearly total number of physicians by sex and ratio of females to males in dermatology, medical nonprocedural, medical procedural, and surgical specialty groups in Ontario, Canada, from 1992 to 2018.

**Figure 2:**
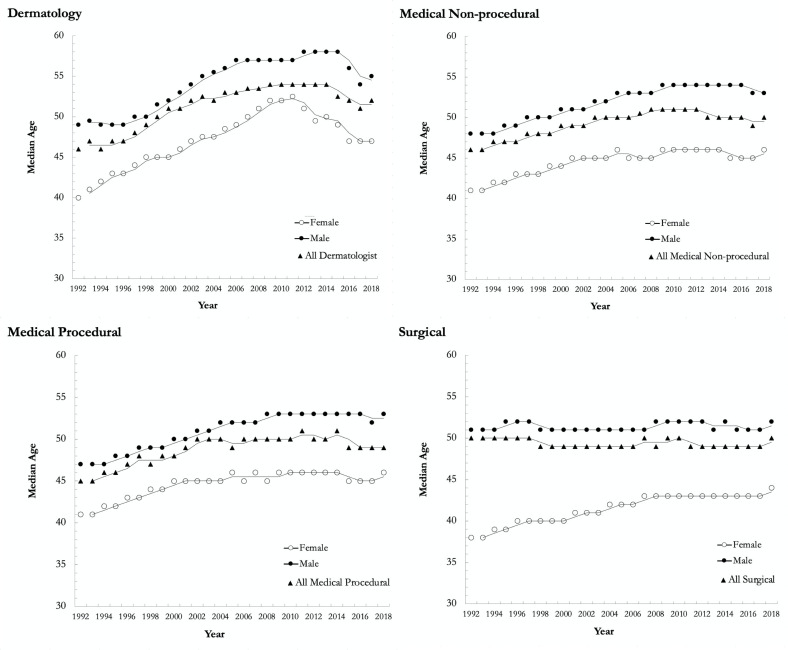
Median age for females, males and overall in dermatology, medical nonprocedural, medical procedural, and surgical specialty groups in Ontario from 1992 to 2018.

A total of 22 389 physicians were included in the database, including 381 dermatologists, 13 004 medical nonprocedural, 1906 medical procedural, and 7098 surgical specialists. The proportion of female dermatologists increased from 32% (*n* = 63) in 1992 to 46% (107) in 2018, compared to 22% (942) to 43% (3,135), 17% (53) to 25% (390), and 9% (242) to 27% (1094) for medical nonprocedural, medical procedural, and surgical specialties, respectively (Figure 1). The median age of female dermatologists in 1992 and 2018 was 40 (interquartile range [IQR]: 36–53) and 47 (IQR: 39–60) years, respectively, compared to 49 (IQR: 42–58) and 55 (IQR: 44–69) years for male dermatologists. An age gap between females and males was seen across all specialty groups, with the greatest difference seen within the surgical specialty group in 1992 (Figure 2). Table 1 summarizes the number, median age, median payments, and practice characteristics of dermatologists and other specialty groups in 2018.

**Table 1:** Number of Physicians, Median Age, Median Payment, and Practice Characteristics for Female and Male Dermatologists in 2018 Compared to Other Specialty Groups.

Reported in Canadian dollars (CAD)	Dermatology *n* = 232		Medical nonprocedural *n* = 7241		Medical procedural *n* = 1556		Surgical *n* = 4014	
	Women	Men	Women	Men	Women	Men	Women	Men
Number of physicians								
All FTEs (%, proportion of total population)	107 (46)	125 (54)	3135 (43)	4106 (57)	390 (25)	1166 (75)	1094 (27)	2920 (73)
<1 FTE (%, proportion of sex population)	46 (43)	46 (37)	1448 (46)	1445 (35)	225 (58)	395 (34)	535 (49)	1065 (36)
1 FTE (%, proportion of sex population)	26 (24)	22 (18)	712 (23)	743 (18)	84 (22)	232 (20)	270 (25)	544 (19)
>1 FTE (%, proportion of sex population)	35 (33)	57 (46)	975 (31)	1918 (47)	81 (21)	539 (46)	289 (26)	1311 (45)
Median age (IQR)								
All FTEs	47 (39, 60)	55 (44, 69)	46 (39, 56)	53 (43, 65)	46 (39, 56)	54 (43, 65)	44 (38, 53)	52 (43, 63)
<1 FTE	44.5 (37, 60)	67.5 (42, 73)	45 (37, 56)	56 (42, 68)	45 (37, 56)	56 (42, 68)	42 (36, 53)	58 (43, 69)
1 FTE	48 (37, 61)	63.5 (48, 70)	46 (39, 55)	51 (42, 64)	46 (38, 55)	51 (42, 64)	45 (39, 53)	52 (43, 62)
>1 FTE	49 (40, 58)	52 (45, 62)	47 (40, 56)	53 (44, 63)	47 (40, 56)	53 (44, 63)	46 (41, 53)	50 (43, 58)
Median payment (IQR)^*^								
All FTEs	282 270 (165 410, 412 310)	342 430 (161 940, 551 730)	226 070 (143 120, 364 650)	316 960 (177 350, 503 260)	227 730 (143 500, 366 500)	317 120 (176 860, 504 800)	353 230 (216 060, 475 310)	459 990 (274 060, 624 960)
<1 FTE	146 290 (93 180, 93 180)	102 660 (66 470, 174 210)	135 450 (77 690, 187 630)	133 360 (63 600, 63 600)	135 450 (78 690, 188 020)	131 520 (63 930, 193 820)	210 470 (117 160, 287 450)	206 360 (104 330, 298 720)
1 FTE	303 910 (281 650, 332 180)	292 280 (275 880, 342 430)	243 930 (206 810, 308 460)	279 910 (214 390, 345 060)	247 500 (207 790, 308 460)	280 190 (214 860, 344 300)	391 990 (362 140, 422 780)	430 230 (391 960, 483 300)
>1 FTE	489 700 (412 310, 627 050)	569 360 (483 960, 765 750)	404 840 (327 730, 515 740)	487 660 (370 910, 702 750)	407 570 (330 530, 518 100)	489 580 (373 140, 703 460)	551 450 (488 990, 666 560)	645 500 (541 000, 810 910)
Median number of patients (IQR)								
All FTEs	2490 (1800, 3860)	3450 (1780, 5740)	650 (270, 145)	1030 (380, 2790)	670 (850, 3490)	1060 (1270, 5710)	1280 (780, 1810)	1530 (880, 2300)
<1 FTE	1910 (1100, 2370)	1420 (890, 2250)	400 (160, 940)	460 (160, 1200)	430 (490, 2120)	490 (460, 2650)	940 (500, 1340)	800 (380, 1390)
1 FTE	2890 (2290, 3610)	3020 (2740, 3630)	740 (330, 1330)	980 (400, 2130)	770 (1110, 3420)	1000 (1370, 4720)	1400 (1020, 1800)	1550 (1140, 2220)
>1 FTE	4560 (2970, 5410)	5960 (4580, 7730)	1130 (510, 2190)	1760 (750, 3890)	1170 (1870, 5400)	1840 (2520, 8270)	1790 (1370, 2350)	2020 (1500, 2950)
Median number of visits (IQR)								
All FTEs	4470 (2820, 6210)	5450 (2840, 9790)	1700 (830, 3350)	5220 (1260, 5600)	7930 (850, 3490)	2780 (1270, 5710)	2910 (1750, 4320)	3460 (1930, 5150)
<1 FTE	3170 (1850, 3760)	2540 (1090, 4060)	960 (480, 2020)	1110 (450, 2600)	980 (490, 2120)	1140 (460, 2650)	2000 (890, 2960)	1590 (600, 2860)
1 FTE	4810 (3960, 5830)	5030 (4280, 5200)	1760 (1080, 3190)	2330 (1350, 4590)	1800 (1110, 3420)	2380 (1370, 4720)	3300 (2430, 4240)	3640 (2660, 4800)
>1 FTE	7180 (5300, 8960)	10530 (8130, 14 830)	3070 (1850, 5220)	4360 (2480, 7930)	3130 (1870, 5400)	4440 (2520, 8270)	4650 (3290, 6130)	4800 (3510, 6510)

Abbreviations: FTE, full-time equivalent; IQR, interquartile range.

^a^Reported in Canadian dollars (CAD).

Regarding the FTE subgroups, the highest proportion of female dermatologists were in the <1 FTE group (43%), while the highest proportion of males were in the >1 FTE group (46%) in 2018. In 1992, dermatologists had the highest ratio of females to males in the 1 FTE (0.6) and >1 FTE groups (0.3), compared to medical nonprocedural (0.4 and 0.1), medical procedural (0.2 and 0.1), and surgical specialty groups (0.1 and 0.04). Among female dermatologists, 49% and 57% were in the ≥1 FTE group in 1992 and 2018, respectively, compared to 66% and 63% of male dermatologists (Table 2).

**Table 2:** Distribution of Females and Males by Full-Time Equivalent Groupings for Dermatology Compared to Other Specialty Groups in 1992 and 2018.

Specialty groupings	Females			Males			Ratio of females to males		
	<1 FTE	1 FTE	>1 FTE	<1 FTE	1 FTE	>1 FTE	<1 FTE	1 FTE	>1 FTE
Dermatology, % (*n*)									
1992	51 (32)	24 (15)	25 (16)	34 (45)	19 (25)	47 (61)	0.7	0.6	0.3
2018	43 (46)	24 (26)	33 (35)	37 (46)	18 (22)	45 (57)	1.0	1.2	0.6
Medical nonprocedural, % (*n*)									
1992	54 (512)	24 (227)	22 (203)	36 (1174)	19 (625)	45 (1483)	0.4	0.4	0.1
2018	46 (1,448)	23 (712)	31 (975)	35 (1445)	18 (743)	47 (1918)	1.0	1.0	0.5
Medical procedural, % (*n*)									
1992	66 (35)	21 (11)	13 (7)	34 (89)	21 (53)	45 (117)	0.4	0.2	0.1
2018	58 (225)	21 (84)	21 (81)	34 (395)	20 (232)	46 (539)	0.6	0.4	0.2
Surgery, % (*n*)									
1992	60 (145)	21 (50)	19 (47)	38 (956)	20 (510)	42 (1054)	0.2	0.1	0.04
2018	49 (535)	25 (270)	26 (289)	36 (1065)	19 (544)	45 (1311)	0.5	0.5	0.2

Abbreviation: FTE, full-time equivalent.

**Figure 3:**
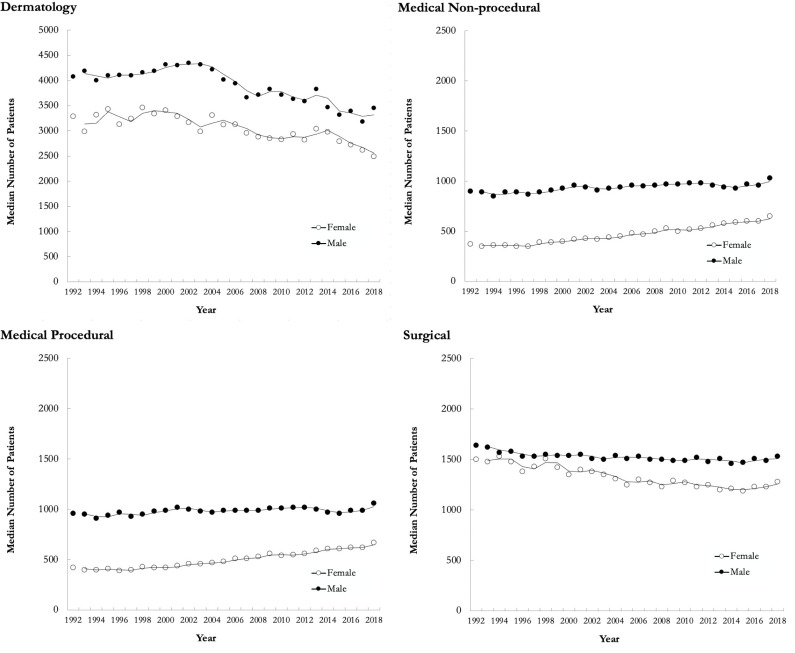
Yearly median number of distinct patients for females and males in dermatology, medical nonprocedural, medical procedural, and surgical specialty groups in Ontario from 1992 to 2018.

Regarding number of distinct patients seen, female dermatologists saw fewer patients across the entire study period compared to their male counterparts, and the number of distinct patients decreased over time for both sexes (Figure 3). From 1992 to 2018, dermatologists on average saw 19% fewer distinct patients. A similar trend was seen with the surgical specialty group, whereby there was a 10% decrease across the study period. Conversely, medical nonprocedural and medical procedural specialties had a 32% and 25% increase in number of distinct patients from 1992 to 2018, respectively. When comparing FTE groups, the ratio of distinct patients for females compared to males increased for the <1 FTE group from 1992 (0.98) to 2018 (1.35). This ratio remained unchanged for the 1 FTE group (0.96, 0.96) and decreased for the >1 FTE group (0.98, 0.77), with female dermatologists seeing 0.77 patients for every 1 patient seen by their male counterparts in 2018. A similar downward trend was seen with median number of visits per patient, where dermatologists had 2.0 visits per patient in 1992 compared to 1.7 in 2018. The 15% decrease in median number of visits per patient from 1992 to 2018 was more than the 4% decrease for surgical specialties (2.4, 2.3), but less than the 32% decrease for medical nonprocedural (3.8, 2.6) and 30% decrease for medical procedural specialties (3.7, 2.6).

**Figure 4:**
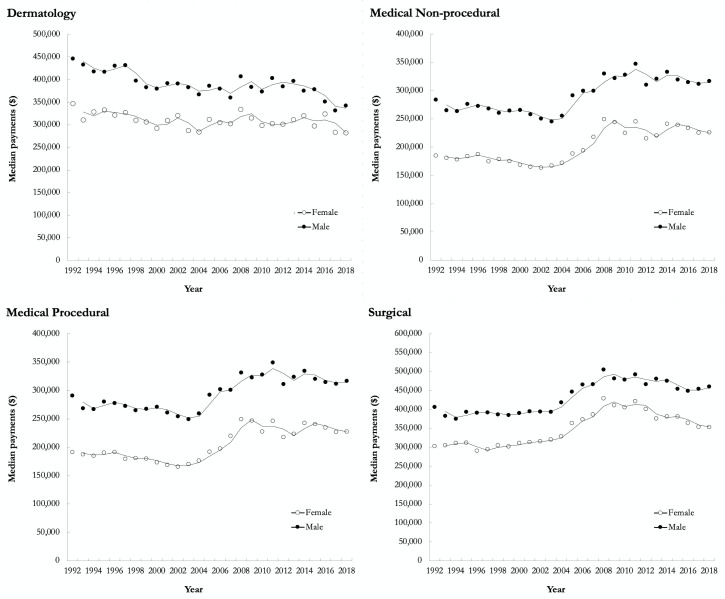
Unadjusted yearly median payments for females and males in dermatology, medical nonprocedural, medical procedural, and surgical specialty groups in Ontario from 1992 to 2018. For all years included in the analysis, the reported dollars were converted to 2018 Canadian dollars.

Regarding billing, dermatologists’ median OHIP payment was $415 340 (IQR: 285 630-566 580) in 1992 compared to $296 750 (IQR: 164 480-493 180) in 2018 (Figure 4). Male dermatologists’ OHIP payments were 20% more than their female counterparts across the entire study period. This was largely driven by the 1 FTE and >1 FTE groups, whereby female dermatologists in the <1 FTE group received 1.21 for every dollar paid from OHIP to males versus 0.99 and 0.90 for the 1 FTE and >1 FTE groups, respectively. In 2018, female dermatologists’ median OHIP payment was 18% ($60 160) less than males, representing the smallest pay gap compared to the 29% ($90 890), 28% ($89 390), and 23% ($106 760) seen in the medical nonprocedural, medical procedural, and surgical specialty groups, respectively (Figure 4). Relative to 1992, the pay gap decreased across all specialty groups; 22% ($99 790), 35% ($99 090), 34% ($99 460), and 26% ($103 900) in the dermatology, medical nonprocedural, medical procedural, and surgical specialty groups, respectively.

**Figure 5:**
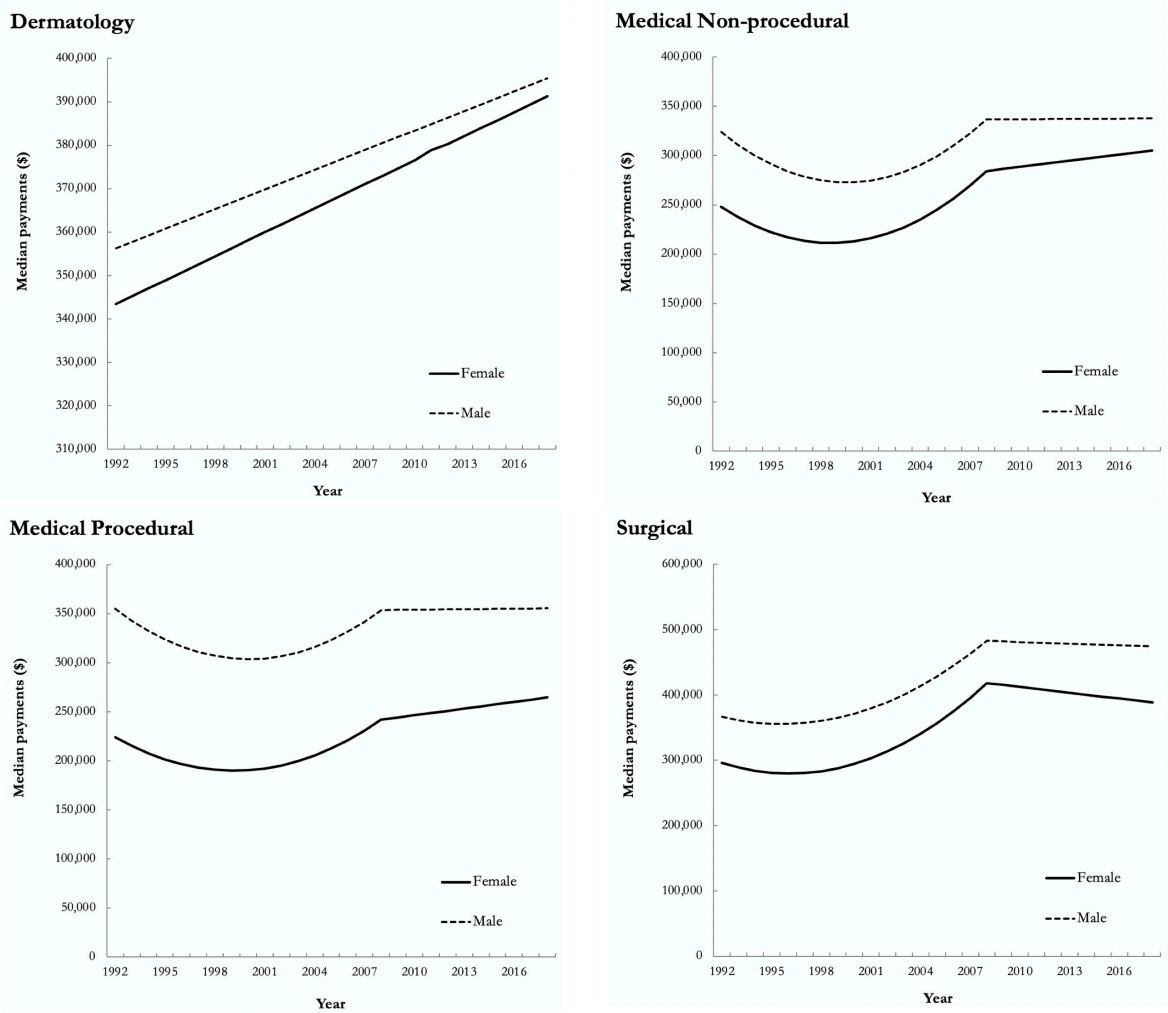
Yearly median payments for females and males in dermatology, medical nonprocedural, medical procedural, and surgical specialty groups in Ontario from 1992 to 2018. Lines depict the predicted median payments obtained from adjusted multiple linear regression models controlling for year, sex-year interaction, age, number of distinct patients seen, number of patient visits, and number of visits per patient. For all years included in the analysis, the reported dollars were converted to 2018 Canadian dollars. Note: The inflection point in 2008 represents the lifting of capitation to physician fees; some physician groups, including dermatology, were exempt from this.

Unadjusted multilevel regression models demonstrated a significant pay gap amongst females and males for all 4 specialty groups (*P* < .001). After adjusting for the number of visits, number of patients, and visits per patient, the sex pay gap remained significant for medical nonprocedural, medical procedural, and surgical specialty groups (*P* < .001), but not for dermatology (*P* = .42), suggesting that the sex pay gaps seen among dermatologists are largely related to sex difference in practice (Figure 5).

## Discussion

We present a population-based study of the changing demographics, practice patterns, and differences in remuneration amongst female and male dermatologists compared to other specialty groups in Ontario, Canada, from 1992 to 2018. Our results highlight the growing female representation in dermatology over the past 3 decades. Notably, the proportion of females has remained relatively high in dermatology since 1992 compared to other specialty groups. In conjunction with increased female representation, there is a growing trend toward increased workload for female dermatologists, with the proportion of ≥1 FTE females increasing from 49% in 1992 to 57% in 2018. Conversely, male workload has remained relatively unchanged; a trend seen across other specialty groups as well.

There has been a notable and steady increase in number of female dermatologists, similar to trends seen across all specialty groups and all levels of training in medicine.^1-3^ A recent publication on trends in representation of female applicants in the Canadian Residency Matching Service (CaRMS) showed that there has been an increasing trend in most, but not all, specialties between 1995 and 2019.^23^ Across the entire study period, 66.5% of the 783 applicants to dermatology were female. Despite the rising female representation, male dermatologists continue to outnumber females who make up 46% of all dermatologists. Similar trends are seen in family medicine with females making up 43% of all practicing family physicians in Ontario.^24^ Despite increasing female representation within dermatology with the proportion of female to male clinicians in 2018 being 0.86, females continue to be under-represented in other specialty groups including medical nonprocedural (76%), medical procedural (33%), and surgical (37%).

It is well established that a sex pay gap exists across many medical specialties.^12-18^ The sex pay gap in dermatology has been previously noted in U.S.-based studies; however, data are often limited to specific populations with limited data on confounding factors that may contribute to sex-based salary gaps.^25-27^ Furthermore, the impact of clinical practice patterns of dermatologists on sex-based pay disparities has not been explored in depth. The results in our study are the first of their kind within dermatology OHIP billings, demonstrating that the sex pay gap can be accounted for after adjusting for number of patients and number of visits. While there has been an increase in proportion of practicing female dermatologists relative to males, this has coincided with a decrease in overall median OHIP payments across the entire study period ($415 340 in 1992 versus $296 750 in 2018; Figure 4). While females billed 20% less than their male colleagues, this can be accounted for by fewer patients and fewer visits per year (Figure 5). This is in contrast to other specialty groups in our analysis, where disparities in median payments for females continued to exist after adjusting for number of visits, number of patients, and visits per patient (Figure 5).^24^ Given that OHIP fee-for-service remuneration for dermatologists is greatly driven by new consultations, decreased OHIP payments may be in part attributed to females seeing fewer patients and more follow-up visits with female dermatologists averaging 1.8 visits per patient compared to 1.6 visits per patient for male dermatologists.

Our findings further support prior speculations that the sex pay gap in medicine is inversely associated with female representation, where an increased proportion of females in a specialty corresponded to a decreased pay gap after considering practice patterns.^4,28,29^ Although the sex pay gap in dermatology can be accounted for by fewer patients and fewer visits per year, patient complexity and time spent with individual patients has not been captured in our dataset. Previous studies have highlighted that female physicians generally spend more time per patient and deal with more issues per visit than males, leading to lower billing in a fee-for-service model.^11,30,31^ As such, overall workload as defined by number of hours spent on direct clinical interaction requires further investigations.

The main strength of this study is that it used data on physician billings collected over 27 years from a single-payer model covering the whole population. However, the study is not without limitations. Firstly, non-OHIP payments were not included (cosmetics, clinical trials, third-party payments, worker’s compensation, etc.). Although the OHIP fee-for-service database captures more than 90% of Ontario physicians who are paid on a fee-for-service basis,^10,32^ services provided by physicians salaried through hospital and community alternate funding plans were not included. In addition, the presented database is of gross payments and does not include overhead costs of running a practice within each of the specialty groups. Lastly, the results of this study are limited to a single province and results may not by fully extrapolated to other fee-for-service healthcare systems. Future studies that explore sex differences while accounting for academic versus community practice, patient complexity, hours devoted to patient care, and use of physician extenders should be explored.

## Conclusion

The landscape of dermatology had changed over the past 3 decades with the proportion of female dermatologists in 2018 being greater than the combined medical and surgical groups. Although there was an overall sex gap in OHIP payments to dermatologists, this difference has decreased over the study period and no longer existed after adjusting for practice volumes. Despite this, proportionately more female dermatologists were <1 FTE and fewer were ≥1 FTE compared to male dermatologists. In pursuit of equity in medicine, the unique challenges and barriers for females in dermatology and other specialties should be further explored.

## References

[1] American Medical Association 2015. Physician Characteristics and Distribution in the U.S. https://www.ama-assn.org/system/files/2019-08/a19-clrpd-report-1.pdf

[bibr2-12034754221119500] Buys YM . Aging and feminization of the physician workforce in Canada: comparing ophthalmologists to all other physicians. Can J Ophthalmol. 2014;49(3):291-296. 10.1016/j.jcjo.2014.03.005 24862778

[3] AAMC . ACGME Residents and Fellows by Sex and Specialty. 2015

[4] Felfeli T. Canizares M. Jin Y-P. Buys YM . Pay Gap among Female and Male Ophthalmologists Compared with Other Specialties. Ophthalmology. 2022;129(1):111-113. 10.1016/j.ophtha.2021.06.015 34271073

[5] Micieli R. Alhusayen R . Changes in the practice patterns and demographics of Ontario dermatologists. J Cutan Med Surg. 2018;22(4):390-399. 10.1177/1203475418762719 29519145

[6] Association of American Medical Colleges 2020. 2020 Fall Applicant, Matriculant, and Enrollment Data Tables. October 2, 2021. https://www.aamc.org/media/49911/download.

[7] Silver JK. Ghalib R. Poorman JA et al. Analysis of gender equity in leadership of Physician-Focused medical specialty societies, 2008-2017. JAMA Intern Med. 2019;179(3):433-435. 10.1001/jamainternmed.2018.5303 30615072 PMC6439704

[8] Hoff T. Lee DR . The gender pay gap in medicine: a systematic review. Health Care Manage Rev. 2021;46(3):E37-E49. 10.1097/HMR.0000000000000290 33534271

[9] Cohen M. Kiran T . Closing the gender pay gap in Canadian medicine. CMAJ. 2020;192(35):E1011-E1017. 10.1503/cmaj.200375 32868274 PMC7458685

[10] Henry D. Schultz S. Glazier R. Bhatia R. Dhalla I. Laupacis A 2012. Payments to Ontario Physicians from Ministry of Health and Long-Term Care Sources, 1992/93 to 2009/10. February 16, 2022.Toronto: Institute for Clinical Evaluative Sciences. https://www.ices.on.ca/Publications/Atlases-and-Reports/2012/Payments-to-Ontario-Physicians

[11] Committee OPHR Ontario Medical Association 2020. Report to Council: Understanding gender pay gaps among Ontario physicians.. Accessed February 16, 2022OMAThoughts. https://www.oma.org/uploadedfiles/oma/media/public/gender-pay-gap-report-august-2020.pdf.

[12] Pelley E. Danoff A. Cooper DS. Becker C . Female physicians and the future of endocrinology. J Clin Endocrinol Metab. 2016;101(1):16-22. 10.1210/jc.2015-3436 26574957

[bibr13-12034754221119500] Mainardi GM. Cassenote AJF. Guilloux AGA. Miotto BA. Scheffer MC . What explains wage differences between male and female Brazilian physicians? A cross-sectional nationwide study. BMJ Open. 2019;9(4):e023811 10.1136/bmjopen-2018-023811 31048423 PMC6502025

[bibr14-12034754221119500] Gilbert SB. Allshouse A. Skaznik-Wikiel ME . Gender inequality in salaries among reproductive endocrinology and infertility subspecialists in the United States. Fertil Steril. 2019;111(6):1194-1200. 10.1016/j.fertnstert.2019.02.004 30922655

[bibr15-12034754221119500] Rosas VGSY. Moscoso-Porras M. Ormeño R. Artica F. Bayes CL. Miranda JJ . Gender income gap among physicians and nurses in Peru: a nationwide assessment. Lancet Glob Health. 2019;7(4):e412-e413. 10.1016/S2214-109X(19)30034-8 30745027

[bibr16-12034754221119500] Okoshi K. Nomura K. Taka F et al. Suturing the gender gap: income, marriage, and parenthood among Japanese surgeons. Surgery. 2016;159(5):1249-1259. 10.1016/j.surg.2015.12.020 26830072

[bibr17-12034754221119500] Desai T. Ali S. Fang X. Thompson W. Jawa P. Vachharajani T . Equal work for unequal pay: the gender reimbursement gap for healthcare providers in the United States. Postgrad Med J. 2016;92(1092):571-575. 10.1136/postgradmedj-2016-134094 27528703

[18] Patel NA. Ji YD. Donoff RB . Clinical productivity and Medicare payments among female and male oral and maxillofacial surgeons. J Oral Maxillofac Surg. 2020;78(5):688-694. 10.1016/j.joms.2019.12.024 32006487

[19] Baker LC . Differences in earnings between male and female physicians. N Engl J Med. 1996;334(15):960-964. 10.1056/NEJM199604113341506 8596598

[20] Canadian Institute for Health Information . Health Care Cost Drivers: The Facts

[21] Henry DA. Schultz SE. Glazier RH. Bhatia RS. Dhalla IA. Laupacis A 2012. Payments to Ontario Physicians from Ministry of Health and Long-Term Care Sources

[22] Henry D. Schultz S. Glazier R. Bhatia R. Dhalla I. Laupacis A . Payments to Ontario Physicians from Ministry of Health and Long-Term Care Sources, 1992/93 to 2009/10Toronto: Institute for Clinical Evaluative Sciences. https://www.ices.on.ca/Publications/Atlases-and-Reports/2012/Payments-to-Ontario-Physicians. Published 2012. Accessed February 16, 2022.

[23] Lorello GR. Silver JK. Moineau G. McCarthy K. Flexman AM . Trends in representation of female applicants and Matriculants in Canadian residency programs across specialties, 1995 to 2019. JAMA Netw Open. 2020;3(11):e2027938. 10.1001/jamanetworkopen.2020.27938 33231640 PMC7686870

[24] Buys YM. Canizares M. Felfeli T. Jin Y . Influence of Age, Sex, and Generation on Physician Payments and Clinical Activity in Ontario, Canada: An Age-Period-Cohort Analysis. Am J Ophthalmol. 2019;197:23-35. 10.1016/j.ajo.2018.09.003 30236775

[25] Sachdeva M. Price KN. Hsiao JL. Shi VY . Gender and RANK salary trends among academic dermatologists. Int J Womens Dermatol. 2020;6(4):324-326. 10.1016/j.ijwd.2020.05.005 33015295 PMC7522812

[bibr26-12034754221119500] Do MH. Lipner SR . Contribution of gender on compensation of Veterans Affairs-affiliated dermatologists: a cross-sectional study. Int J Womens Dermatol. 2020;6(5):414-418. 10.1016/j.ijwd.2020.09.009 33898710 PMC8060668

[27] Guss L. Chen Q. Hu C. Guss Z. Kang S. Grossberg A . Income inequality between male and female clinical faculty at public academic dermatology departments. J Am Acad Dermatol. 2020;83(2):633-636. 10.1016/j.jaad.2019.10.045 31678466

[28] Greenberg CC . Association for academic surgery presidential address: sticky floors and glass ceilings. J Surg Res. 2017;219:ix-0. 10.1016/j.jss.2017.09.006 29078918

[29] Gambhir S. Daly SC. Elfenbein D et al. The effect of transparency on the gender-based compensation gap in surgical disciplines within a large academic healthcare system. Surg Endosc. 2021;35(6):2607-2612. 10.1007/s00464-020-07679-1 32488656

[30] Hedden L. Barer ML. Cardiff K. McGrail KM. Law MR. Bourgeault IL . The implications of the feminization of the primary care physician workforce on service supply: a systematic review. Hum Resour Health. 2014;12(1):32 10.1186/1478-4491-12-32 24898264 PMC4057816

[31] Roter DL. Hall JA. Aoki Y . Physician gender effects in medical communication: a meta-analytic review. JAMA. 2002;288(6):756-764. 10.1001/jama.288.6.756 12169083

[32] Schultz S. Glazier R. Graves E. Schull M. Sutradhar R . Payments to Ontario physicians from Ministry of health and long-term care sources: update 2005/06 to 2017/18. Toronto: Institute for Clinical Evaluative Sciences. 2022 https://www.ices.on.ca/~/media/Files/AHRQ/AHRQ-Reports/Physician-Compensation-Update-2005_06-to-2017_18.ashx. Published 2019.

